# Unmet Need for Family Planning and Experience of Unintended Pregnancy Among Female Sex Workers in Urban Cameroon: Results From a National Cross-Sectional Study

**DOI:** 10.9745/GHSP-D-19-00330

**Published:** 2020-03-30

**Authors:** Anna L. Bowring, Sheree Schwartz, Carrie Lyons, Amrita Rao, Oluwasolape Olawore, Iliassou Mfochive Njindam, Jimmy Nzau, Ghislaine Fouda, Guy H. Fako, Gnilane Turpin, Daniel Levitt, Sandra Georges, Ubald Tamoufe, Serge C. Billong, Oudou Njoya, Anne-Cécile Zoung-Kanyi, Stefan Baral

**Affiliations:** a Department of Epidemiology, Johns Hopkins Bloomberg School of Public Health, Baltimore, MD, USA.; b Burnet Institute, Melbourne, Australia.; c Metabiota, Yaounde, Cameroon.; d CARE SRHR Global Team, Atlanta, GA, USA.; e CARE Cameroon, Yaounde, Cameroon.; f CARE USA, New York, NY, USA.; g Johns Hopkins Cameroon Program, Yaounde, Cameroon.; h Faculty of Medicine and Biomedical Sciences, University of Yaoundé I, Yaounde, Cameroon.; i Division of Operations Research, Ministry of Health, Yaounde, Cameroon.

## Abstract

**Background:**

Female sex workers (FSWs) in Cameroon commonly have unmet need for contraception posing a high risk of unintended pregnancy. Unintended pregnancy leads to a range of outcomes, and due to legal restrictions, FSWs often seek unsafe abortions. Aside from the high burden of HIV, little is known about the broader sexual and reproductive health of FSWs in Cameroon.

**Methods:**

From December 2015 to October 2016, we recruited FSWs aged ≥18 years through respondent-driven sampling across 5 Cameroonian cities. Cross-sectional data were collected through a behavioral questionnaire. Modified-robust Poisson regression was used to approximate adjusted prevalence ratios (aPR) for TOP and current use of effective nonbarrier contraception.

**Results:**

Among 2,255 FSWs (median age 28 years), 57.6% reported history of unintended pregnancy and 40.0% reported prior TOP. In multivariable analysis, TOP history was associated with current nonbarrier contraceptive use (aPR=1.23, 95% confidence interval [CI]=1.07, 1.42); ever using emergency contraception (aPR=1.34, 95% CI=1.17, 1.55); >60 clients in the past month (aPR=1.29, 95% CI= 1.07, 1.54) compared to ≤30; inconsistent condom use with clients (aPR=1.17, 95% CI=1.00, 1.37); ever experiencing physical violence (aPR=1.24, 95% CI=1.09, 1.42); and older age. Most (76.5%) women used male condoms for contraception, but only 33.2% reported consistent condom use with all partners. Overall, 26.4% of women reported currently using a nonbarrier contraceptive method, and 6.2% reported using a long-acting method. Previous TOP (aPR=1.41, 95%CI=1.16, 1.72) and ever using emergency contraception (aPR=2.70, 95% CI=2.23, 3.26) were associated with higher nonbarrier contraceptive use. Recent receipt of HIV information (aPR=0.72, 95% CI=0.59, 0.89) and membership in an FSW community-based organization (aPR=0.73, 95% CI=0.57, 0.92) were associated with lower use nonbarrier contraceptive use.

**Conclusions:**

Experience of unintended pregnancies and TOP is common among FSWs in Cameroon. Given the low use of nonbarrier contraceptive methods and inconsistent condom use, FSWs are at risk of repeat unintended pregnancies. Improved integration of client-centered, voluntary family planning within community-led HIV services may better support the sexual and reproductive health and human rights of FSWs consistent with the United Nations Declaration of Human Rights.

## Abstract (precis)

Female sex workers (FSWs) in Cameroon have unmet need for effective contraception, and experience of unintended pregnancy and pregnancy termination is common. Reducing barriers to accessing high-quality, voluntary family planning services in FSW-focused community services is a key strategy to promote client-centered care, promote informed choice, reduce unintended pregnancies, and improve quality of life for FSWs.

## BACKGROUND

Female sex workers (FSWs) have a disproportionate burden of HIV infection and experience systematic barriers to accessing existing HIV prevention and treatment services.^1^ Based on 9 studies conducted during 2011–2016, HIV prevalence among women who sell sex ranged from 11% to 24% across western and central Africa and was more than 60% across southern Africa.^2^ Specific guidelines for HIV interventions among FSWs have shaped programs targeting HIV prevention, testing, and treatment. However, less attention has been given to FSWs' other comprehensive sexual and reproductive health services, including voluntary family planning.

FSWs may consider pregnancy prevention a more influential motivator for condom use than HIV prevention.^3^ Previous work has demonstrated that unintended pregnancy is a high-priority issue for FSWs, with unintended pregnancy incidence of 27 per 100 person-years among FSWs in low- and middle-income settings without presence of a sexual and reproductive health intervention.^4^ One potential outcome of unintended pregnancy is termination. Among FSWs, experience of termination of pregnancy (TOP) is widespread in global estimates and often higher than national estimates among all women.^5^ In many countries where FSWs are most affected by unintended pregnancy, abortion policy is generally restrictive,^6^ which can lead to women seeking unsafe abortions. Across sub-Saharan Africa specifically, it is estimated that unsafe abortions contribute to at least 10% of maternal deaths.^6^^–^^8^ In addition, unintended pregnancy leading to live births can have financial implications that may perpetuate vulnerability and HIV risks related to sex work.^9^^,^^10^

Male and female condoms remain important means of preventing unintended pregnancy and HIV and STIs among FSWs in 2019.^11^ However, extensive social and structural factors affect both condom use and condom failure among FSWs.^12^^–^^15^ These factors include punitive laws and policies, local policing practices, economic insecurity, intersecting stigmas, physical and sexual violence, and drug and alcohol use during sex work.^16^ Even in settings with high levels of consistent condom use with paying partners, condom use between FSWs and their nonpaying or emotional partners is low due to interpersonal barriers to condom use.^17^^,^^18^ Previous studies have demonstrated associations between having regular, emotional partners and unintended pregnancy or abortion.^19^^,^^20^

In light of the reduced effectiveness of condoms with typical use as opposed to perfect use,^21^^,^^22^ especially in the context of violence,^16^ and differences in condom use with clients and nonpaying partners, dual-method contraceptive use—condoms combined with an effective nonbarrier method—for both HIV/STI and pregnancy prevention is recommended for key cisgender women populations.^23^ However, widespread studies have shown that FSWs across several low- and middle-income countries have significant unmet need for effective contraception and voluntary family planning services.^5^^,^^24^ The current use of modern nonbarrier methods among FSWs is estimated to be below 40% in numerous settings across sub-Saharan Africa,^25^^–^^27^ and FSWs report most often using injectables or oral contraceptive pills.^14^^,^^26^^,^^28^^,^^29^ Although these are both effective methods with perfect use,^21^ they are user-dependent, are prone to misuse due to delays in renewal or well-documented issues with daily adherence, respectively,^28^ and often have high discontinuation of use due to side effects and barriers to accessing family planning services.^30^^–^^32^ Long-acting reversible contraceptives (LARCs), which include intrauterine devices (IUDs) and implants, are globally recommended for reducing unintended pregnancy due to high effectiveness, continuation rates, and higher tolerability compared to other nonbarrier methods,^31^ but availability and utilization of LARCs remain low among FSWs in much of sub-Saharan Africa.^4^^,^^33^

Women in Cameroon most commonly use short-acting and user-dependent contraceptive methods. In 2015, among a household survey of women in urban Yaoundé using any contraceptive method, only 8% were using LARCs and an additional 5% reported using an oral contraceptive pill.^34^ Perceived efficacy and easy accessibility were the 2 main considerations for contraceptive choice, and only half of women were aware of LARCs. In Cameroon, LARCs are intended to be available through hospitals and selected clinics that provide family planning and reproductive health services.^34^^,^^35^ However, a recent evaluation indicates that stock-outs prevail, with 70%–80% of facilities reporting stock-outs of each offered LARC method on the day of assessment.^36^ The unmet need for contraception results in high numbers of unintended pregnancies.^36^

Cameroon's abortion policy has been described as restrictive: legally permitted only in cases of rape, incest, or to preserve a woman's physical or mental health, and even then, it can be difficult to obtain legal standing for abortion.^37^ Nonetheless, experiences of abortion are common, with estimates ranging from 20%–35% prevalence among women.^38^^–^^40^ Due to legal and regulatory restrictions, abortions are often unsafe. Notably, unsafe abortions have been demonstrated to account for one-quarter of registered maternal deaths in Cameroon.^41^^,^^42^ Although generalizable data are limited, abortions have been reported in both home and clinical settings and are most commonly provided by nurses, general practitioners, a friend, or through self-induction. The most commonly reported procedures are dilatation and curettage, manual vacuum aspiration, or misoprostal.^43^ There is no standard practice for postabortion care in Cameroon, but it may include further manual vacuum aspiration, sharp curettage, or misoprostal depending on resource availability.^37^^,^^44^ It is not clear whether postabortion care services routinely include family planning counseling and methods.^37^

As of 2016, more than 1 in 4 sex workers in Cameroon are estimated to be living with HIV^12^ compared to around 1 in 20 among all women aged 15–64 years.^45^ Sex work is illegal in Cameroon, and arrests as well as police violence, raids, and extortion have been commonly reported.^46^^,^^47^ Earlier work has demonstrated that physical and sexual violence as well as depression are prevalent among FSWs in Cameroon and associated with condom nonuse, being offered more money for condomless sex, and condom failure.^46^^,^^48^ These circumstances may similarly affect risk of unintended pregnancy. However, there are limited studies evaluating unintended pregnancy, pregnancy outcomes, or related consequences with the majority of studies focused on HIV risks. In response, these analyses aim to fill a gap in knowledge of reproductive health among FSWs in Cameroon by characterizing the prevalence of unintended pregnancy, TOP, and contraceptive use.

These analyses aim to fill a gap in knowledge of reproductive health among FSWs in Cameroon.

## METHODS

### Study Overview and Population

This is a secondary analysis of data collected through a cross-sectional respondent-driven sampling (RDS) study in 5 cities of Cameroon: Bamenda, Bertoua, Douala, Kribi, and Yaoundé. These represent the 2 largest cities and cities with high absolute and relative population of sex workers.^49^ This study was conducted from December 2015 to October 2016 as a baseline assessment to inform service provision models for the Continuum of prevention, care, and treatment of HIV/AIDS with Most at-risk Populations (CHAMP) program, implemented by an alliance of community-based organizations (CBOs) and led by the nongovernmental organization CARE.

Women were eligible to participate if they were assigned the female sex at birth, reported sex work as their principal source of income in the previous year, were 18 years or older, spoke French or English, and had resided in the city of recruitment for at least 3 months before recruitment.

### Study Recruitment and Procedures

Methods have been reported in more detail previously.^12^^,^^50^ Briefly, 6 FSWs were purposively selected as “seeds” based on their peer networks. Seeds and subsequent participants were provided with referral coupons to recruit up to 3 other FSWs from their social network for participation. Sampling continued until recruitment across cities reached the desired sample size (Bamenda: 340, Bertoua: 301, Douala: 460, Kribi: 578, Yaoundé: 571). Sample size calculations were based on the estimated HIV prevalence by region and included a design effect of 2, alpha of 0.01, and were intended to measure HIV prevalence with a precision of ±3%.

Trained interviewers assessed participant eligibility, obtained informed verbal consent, and administered a 45–60-minute questionnaire on individual, community, network, and structural-level HIV risks; sexual and reproductive health; and service engagement. Questionnaires and biological data were linked by a confidential unique identifying code, and data were nonidentifiable. Women diagnosed with HIV were supported for linkage to treatment; others were linked to community-based HIV prevention and support services. All participants were reimbursed 2,000 FCFA (US$4) and received an additional 1,000 FCFA (US$2) for each successful recruit.

Ethical approval and administrative clearance were obtained from the Cameroonian National Research Ethics Committee (reference 2015/05/591/CE/CNERSH/SP and 2016/06/782/CE/CNERSH/SP) and Ministry of Public Health (reference 631 2315), respectively.

### Outcomes

The 2 primary outcomes of interest were lifetime experience of TOP and current use of a nonbarrier contraceptive. Given the complexity of measuring unintended pregnancy and bias associated with retrospective reporting of pregnancy intentions,^51^ TOPs was used as an indirect measure of unintended pregnancy as well as an indicator of potential risk to women's health due to unsafe TOP.^8^ Respondents were asked the number of lifetime pregnancies experienced followed by whether they had ever experienced the following outcomes: live birth, stillbirth, miscarriage, or voluntary termination. Participants were also asked to select all methods currently being used to prevent pregnancy. Nonbarrier contraceptive use considered current use of an oral contraceptive pill, IUD, injectable method, implant, or female sterilization. Secondary outcomes considered were unintended pregnancy, defined as ever having a self-reported an unplanned or unwanted pregnancy and LARC use. Unintended pregnancy was defined as a pregnancy that was unplanned or unwanted. LARC use included current use of a contraceptive implant or IUD.

### Covariates of Interest

The association of multiple covariates with TOP and nonbarrier contraception were considered. Covariates related to service access included previous HIV testing, receipt of information on HIV in the previous 6 months, and member of a CBO working with FSWs.

HIV status was categorized as HIV-negative, HIV-positive previously diagnosed, HIV-positive newly diagnosed, and indeterminate based on serological testing and self-reported HIV status.

Covariates related to other contraceptive use and sexual behavior included ever using emergency contraception, number of clients in the past month, condom use with clients, condom use with regular nonpaying partners, number of regular nonpaying partners in the past year, number of times experienced condom failure in the past year, and whether engaged in sex work during last pregnancy. Consistency of condom use was assessed among participants reporting at least 1 of given partner type on an average week and was assessed separately for regular and casual paying and nonpaying partners. Condom use was defined as consistent if they always reported using condoms during vaginal and anal sex with the given partner type and inconsistent if they reported using condoms less than always. In regression analysis, categorization of condom use with clients (regular and casual nonpaying partners combined) also included not having vaginal or anal sex with clients. In addition, future pregnancy intentions were considered, defined as intending to have children in the future (yes, no, does not know).

Covariates related to structural determinants of health included ever experiencing physical violence or assault, ever being forced to have sex, and experience of health-related stigma. Health-related stigma included reporting any of the following in relation to involvement in sex work: ever being afraid of seeking health services, avoidance of health services, mistreatment in a health center, heard health providers gossip, denied health services, or forced to have an HIV test.

### Analytical Approach

Data were combined from 5 independent study sites. Primary outcomes are presented as both crude and RDS-adjusted proportions, the latter were calculated by study site and averaged to estimate overall RDS-adjusted proportion.

On account of primary outcomes being common, modified Poisson regression with robust variance was used to approximate prevalence ratios (PR) for each primary outcome.^52^^,^^53^ Models were corrected for RDS-weighting and clustering by seed. Firstly, bivariate associations were assessed for variables with theoretical association to the given outcomes. A multivariable model was developed for each primary outcome with variables with a *P* value <.1 in bivariate association. Any regular nonpaying sex partner(s) in the past year and consistent condom use with clients were included *a priori* in both models, and age group was included *a priori* when modelling associations with history of TOP. To prevent collinearity between HIV testing history and previous HIV diagnosis, only HIV status was considered in multivariable models.

Site-specific RDS sampling weights were computed using the Gile's SS estimator, which generates weight by estimating the probability of being included in the study using self-reported network size, using RDS Analyst (version 0.42, Los Angeles, CA). All other analyses were conducted using Stata version 14 (StataCorp, College Station, TX). Unless indicated, missing data were omitted from analyses.

## RESULTS

In total, 2,255 FSWs were recruited, age range 18–70 years (median age 28 years, interquartile range [IQR] 23–36) (Table 1). The median age of first sex in exchange for money or goods was 22 years (IQR 12–50). The majority of women (77.0%, 1,735/2,254) were never married but reported at least 1 regular nonpaying partner in the previous year (72.8%, 1,638/2,252).

**TABLE 1.** Sociodemographic Characteristics of Female Sex Workers in 5 Cities, Cameroon

	N^a^	n(%)
Age group, years		
18-24	2,255	724 (32.1)
25-34	2,255	890 (39.5)
35+	2,255	641 (28.4)
Education level		
Primary school or less	2,254	702 (31.1)
Any secondary education or higher	2,254	1,552 (68.9)
Monthly income^b^		
<50 000 XAF	2,247	663 (29.5)
>=50,000 XAF	2,247	1,584 (70.5)
Age first sold sex for money or goods, years		
<18	2,234	297 (13.3)
18-24	2,234	1,038 (46.5)
25+	2,234	899 (40.2)
Number of clients in past month		
0–10	2,255	681 (30.2)
11–30	2,255	518 (23.0)
31–50	2,255	238 (10.6)
51+	2,255	723 (32.1)
Unknown	2,255	95 (4.2)
Marital status		
Never married	2,254	1,735 (77.0)
Married	2,254	22 (1.0)
Stable partner	2,254	233 (10.3)
Separated/divorced/widowed	2,254	264 (11.7)
Number of regular nonpaying partners in past year		
None	2,252	614 (27.3)
One	2,252	1,222 (54.3)
2+	2,252	416 (18.5)

a Minor changes in denominator due to missing data.

b Income through formal and informal work with a threshold of 50,000 XAF (∼US$90) per month based on national minimum wage (36,270 XAF/month).

### Reproductive Experience

Overall, 91.5% (2,058/2,250) of women reported ever being pregnant, and 57.6% (1,294/2,248) of women reported ever having an unintended pregnancy (Table 2). Most women (83.9%, 1,892/2,255) reported having living biological children.

**TABLE 2.** Reproductive Experience and Contraceptive Use of Female Sex Workers in 5 Cities, Cameroon

	N	n (%)
Number of lifetime pregnancies		
0	2,250	192 (8.5)
1–2	2,250	800 (35.6)
3–4	2,250	634 (28.2)
5+	2,250	624 (27.7)
Ever had an unintended pregnancy	2,248	1,294 (57.6)
Number of living biological children		
0	2,255	363 (16.1)
1	2,255	624 (27.7)
2	2,255	587 (26.0)
3+	2,255	681 (30.2)
Among FSWs ever pregnant:		
Any live births	2,058	1,915 (93.1)
Any still births	2,058	164 (8.0)
Any miscarriage	2,058	447 (21.7)
Any termination of pregnancy	2,058	902 (43.8)
Sought antenatal care at last pregnancy	2,057	1,712 (83.2)
Among FSWs with any live births (N=1915)		
FSW age at first birth		
<18 years	1,891	1,286 (68.0)
18+ years	1,891	605 (32.0)
Births while FSW		
0	1,892	1,235 (65.3)
1	1,892	405 (21.4)
2+	1,892	252 (13.3)
Engaged in sex work during last pregnancy	1,903	650 (34.2)
Time to return to sex work during last pregnancy		
0–3 months	634	311 (49.0)
4–6 months	634	143 (22.6)
7–9 months	634	66 (10.4)
10–12 months	634	78 (12.3)
>1 year	634	36 (5.7)
Future pregnancy intentions		
No	2,255	761 (33.7)
Yes	2,255	1452 (64.4)
Don't know	2,255	42 (1.9)
Consistent condom use^a^:		
With clients	2,237	1734 (77.5)
With regular nonpaying partners	1,565	306 (19.6)
With all partners^b^	2,251	747 (33.2)
Current contraceptive use^c^:		
Male condom	2,255	1,724 (76.5)
Female condom	2,255	697 (30.9)
Oral contraceptive pill	2,255	284 (12.6)
Injectable	2,255	205 (9.1)
Intrauterine device	2,255	52 (2.3)
Implant	2,255	93 (4.1)
Diaphragm	2,255	2 (0.1)
Rhythm method	2,255	511 (22.7)
Withdrawal method	2,255	224 (9.9)
Female sterilization	2,255	32 (1.4)
Other^d^	2,255	62 (2.7)
Ever used emergency contraception	2,255	355 (15.8)

Abbreviations: FSW, female sex worker.

a Reported among individuals reporting at least 1 of given partner type in an average week and refers to condom use during vaginal and anal sex.

b Incudes regular and casual paying partners and regular and casual nonpaying partners.

c Respondents could select more than one contraceptive and percentages do not add up to 100.

d Includes drinking whisky (n=22), traditional medicine (n=8), salted water (n=7), paracetamol/aspirin/quinine (n=5), menopause (n=5), Nescafé (n=3).

Among women with any pregnancy experience, 93.1% (1,915/2,058) had any live births, and 32.3% (657/2,035) of women reported any births since beginning to sell sex. At last pregnancy 34.2% (650/1,903) of women concurrently engaged in sex work.

Overall, 64.4% (1,452/2,255) women intended to become pregnant in the future. The most common contraceptive methods reported being currently used were male condoms (76.5%), female condoms (30.9%), and the rhythm method (22.7%); more than 1 response was permissible.

Based on lifetime recall, 15.8% (355/2,255) of women reported ever using emergency contraception. Among these women, the median times of emergency contraception use was 7 (IQR 2–34).

### TOP Experience

Among all women, 40.0% (902/2,250) reported a TOP (RDS-adj 39.8%, [35.1%, 44.7%]) (Table 3). TOP was higher among women living with HIV (44.3%, 243/548) than those who were not living with HIV (38.8%, 658/1,695, *P*=.027).

**TABLE 3.** Crude and RDS-Adjusted Prevalence of TOP and Current Nonbarrier Contraceptive Method Use by Study Site and Overall

	Lifetime Experience of TOP					Current Use of a Nonbarrier Contraceptive Method				
	Crude			RDS-Adjusted		Crude			RDS-Adjusted	
	N	n	%	%	(95% CI)	N	n	%	%	(95% CI)
Yaoundé	573	219	38.2	38.6	(34.4, 42.9)	574	153	26.7	27.3	(23.5, 31.5)
Douala	457	233	51.0	50.6	(45.7, 55.5)	457	91	19.9	18.6	(15.2, 22.6)
Bertoua	300	81	27.0	26.8	(22.0, 32.2)	304	89	29.3	30.1	(25.0, 35.7)
Bamenda	341	169	49.6	48.5	(42.9, 54.2)	341	117	34.3	34.2	(29.0, 39.8)
Kribi	579	200	34.5	34.5	(30.7, 38.5)	579	146	25.2	25.3	(21.9, 29.0)
Overall	2,250	902	40.1	39.8	(35.1, 44.7)	2,255	596	26.4	27.1	(22.9, 31.7)

Abbreviations: CI, confidence interval; RDS, respondent-driven sampling; TOP, termination of pregnancy.

In multivariable analyses, variables associated with higher prevalence of lifetime TOP were: older age, aged 25-34 years (aPR=1.46, 95% confidence interval [CI]=1.19, 1.79) or ≥35 years (aPR=1.70, 95% CI=1.35, 2.13) compared to aged 18–24 years; residing in Douala (aPR=1.21, 95% CI=1.04, 1.42) or Bamenda (aPR=1.26, 95% CI=1.04, 1.53) compared to Yaoundé; currently using a nonbarrier contraceptive (aPR=1.23, 95% CI=1.07, 1.42); ever using emergency contraception (aPR 1.34, 95% CI=1.17, 1.55); >60 clients in the past month (aPR 1.29, 95% CI=1.07, 1.54) compared to 30 or less; reporting inconsistent condom use with clients (aPR=1.17, 95% CI=1.00, 1.37); and ever experiencing physical violence or assault (aPR=1.24, 95% CI=1.09, 1.42) (Table 4).

**TABLE 4.** Associations with Experience of TOP Among FSWs in Univariate and Multivariable Poisson Regression Corrected for RDS-Weighting and Clustering by Seed, Cameroon

	N	TOP		Unadjusted Analysis			Adjusted Analysis		
		n	%	PR	95% CI	*P* Value	aPR	95% CI	*P* Value
Site									
Yaounde	573	219	38.2	REF			REF		
Douala	457	233	51.0	1.31	(1.13, 1.52)	<.001	1.21	(1.04, 1.42)	.01
Bertoua	300	81	27.0	0.70	(0.56, 0.87)	.001	0.87	(0.69, 1.10)	.26
Bamenda	341	169	49.6	1.26	(1.07, 1.48)	.005	1.26	(1.04, 1.53)	.02
Kribi	579	200	34.5	0.89	(0.76, 1.05)	.17	1.04	(0.88, 1.23)	.66
Age group, years									
18–24	722	203	28.1	REF			REF		
25–34	889	388	43.6	1.58	(1.29, 1.93)	<.001	1.46	(1.19, 1.79)	<.001
35+	639	311	48.7	1.73	(1.42, 2.12)	<.001	1.70	(1.35, 2.13)	<.001
Education level									
Primary school or less	700	272	38.9	REF					
Any secondary education or higher	1,549	630	40.7	1.01	(0.88, 1.17)	.86			
Previously tested for HIV^a^									
No	223	49	22.0	REF					
Yes	2024	851	42.0	1.92	(1.32, 2.79)	.001			
HIV status									
Negative	1,695	658	38.8	REF			REF		
Positive, previously diagnosed	290	148	51.0	1.31	(1.16, 1.49)	<.001	1.13	(0.96, 1.34)	.13
Positive, newly diagnosed	258	95	36.8	0.95	(0.80, 1.13)	.54	0.88	(0.71, 1.10)	.27
Indeterminate	7	1	14.3	0.37	(0.06, 2.26)	.28	1.82	(0.77, 4.30)	.17
Received any information on HIV in past 6 months									
No	755	268	35.5	REF			REF		
Yes	1,495	634	42.4	1.21	(1.04, 1.41)	.01	1.13	(0.96, 1.31)	.13
Uses nonbarrier contraceptive									
No	1,655	619	37.4	REF			REF		
Yes	595	283	47.6	1.29	(1.12, 1.48)	<.001	1.23	(1.07, 1.42)	.003
Ever used emergency contraception									
No	1,892	705	37.3	REF			REF		
Yes	354	196	55.4	1.36	(1.18, 1.57)	<.001	1.34	(1.17, 1.55)	<.001
Engaged in sex work during last pregnancy									
No	1,253	549	43.8	REF					
Yes	650	268	41.2	0.90	(0.77, 1.05)	.19			
Intend to have children in the future									
No	759	335	44.1	1.16	(1.02, 1.33)	.03	REF		
Yes	1,449	547	37.8	REF			1.01	(0.87, 1.18)	.86
Does not know	42	20	47.6	1.00	(0.55, 1.81)	.99	0.93	(0.54, 1.59)	.79
Number of clients in past month									
0–30	1195	450	37.7	REF			REF		
31–60	346	138	39.9	1.10	(0.91, 1.34)	.32	1.12	(0.91, 1.38)	.27
>60	615	289	47.0	1.27	(1.09, 1.48)	.002	1.29	(1.07, 1.54)	.006
Unknown	94	25	26.6	0.88	(0.53, 1.45)	.61	1.01	(0.57, 1.78)	.98
Condom use during vaginal/anal sex with clients									
Consistent	1745	688	39.4	REF			REF		
Inconsistent	502	213	42.4	1.09	(0.92, 1.28)	.33	1.17	(1.00, 1.37)	.04
Any regular sex partner(s) in past year									
No	611	232	38.0	REF			REF		
Yes	1,636	669	40.9	1.14	(0.97, 1.33)	.11	1.15	(0.99, 1.34)	.07
Condom failure in the past year									
Never	875	322	36.8	REF					
1–4 times	803	337	42.0	1.07	(0.92, 1.25)	.40			
5+ times	515	226	43.9	0.98	(0.82, 1.16)	.79			
Ever experienced physical violence or assault									
No	1688	626	37.1	REF			REF		
Yes	558	276	49.5	1.27	(1.11, 1.46)	.001	1.24	(1.09, 1.42)	.001
Ever forced to have sex									
No	1515	576	38.0	REF					
Yes	733	325	44.3	1.09	(0.94, 1.25)	.25			

Abbreviations: aPR, adjusted prevalence ratio; CI, confidence interval; FSW, female sex worker; PR, prevalence ratio; RDS, respondent-driven sampling; TOP, termination of pregnancy.

a Excluded from adjusted analyses due to collinearity with HIV status.

### Condom Use

Overall, 77.5% (1,734/2,237) of women reported consistent condom use with clients. In addition to paying clients, FSWs reported nonpaying regular (72.7%, 1,638/2,252) and casual (22.6%, 509/2,252) partners in the previous year. Considering all sex partners, 33.2% (747/2,251) of women reported consistent condom use (Table 2).

In the past year, 36.7% (806/2,198) of participants reported experiencing condom failure 1–4 times and a further 23.5% (516/2,198) 5 or more times.

### Nonbarrier Contraceptive Use

Overall, 6.2% (140/2,255) women reported currently using any LARC, and 26.4% (596/2,255 RDS-adj 27.1% [22.9%, 31.7%]) reported currently using any nonbarrier contraceptive method (Table 3). Nonbarrier contraception was trending higher among women not living with HIV (27.6%, 469/1,698) compared to women living with HIV (22.9%, 126/550, *P*=.072). Approximately half (51.2%, 1,152/2,251) of women reported either nonbarrier contraceptive use or consistent condom use with all partners.

In multivariable analyses, variables associated with higher use of nonbarrier contraception were: previous TOP (aPR=1.41, 95% CI=1.16, 1.72) and ever using emergency contraception (aPR=2.70, 95% CI=2.23, 3.26). Variables associated with lower use of nonbarrier contraception were: receipt of HIV information in the previous 6 months (aPR=0.72, 95% CI=0.59, 0.89) and membership in an FSW-CBO (aPR=0.73, 95% CI=0.57, 0.92) (Table 5).

**TABLE 5.** Associations with Nonbarrier Contraceptive Method Use Among FSWs in Univariate and Multivariable Poisson Regression Corrected for RDS-Weighting and Clustering by Seed, Cameroon

	N	Uses Nonbarrier Contraceptive Method		Unadjusted Analysis			Adjusted Analysis		
		n	%	PR	95% CI	*P* Value	aPR	95% CI	*P* Value
Site									
Yaounde	574	153	26.7	REF			REF		
Douala	457	91	19.9	0.68	(0.53, 0.87)	.002	0.84	(0.67, 1.07)	.16
Bertoua	304	89	29.3	1.10	(0.88, 1.39)	.41	1.21	(0.96, 1.53)	.10
Bamenda	341	117	34.3	1.25	(1.01, 1.55)	.04	1.15	(0.91, 1.44)	.24
Kribi	579	146	25.2	0.93	(0.76, 1.13)	.46	1.03	(0.84, 1.27)	.78
Age group (years)									
18–24	724	169	23.3	REF					
25–34	890	275	30.9	1.11	(0.87, 1.43)	.40			
35+	641	152	23.7	0.85	(0.64, 1.13)	.27			
Education level									
Primary school or less	702	175	24.9	REF			REF		
Any secondary education or higher	1,552	420	27.1	1.39	(1.10, 1.76)	.005	1.21	(0.97, 1.50)	.09
HIV status									
Negative	1,698	469	27.6	REF			REF		
Positive, previously diagnosed	290	66	22.8	0.65	(0.47, 0.90)	.009	0.85	(0.62, 1.18)	.33
Positive, newly diagnosed	260	60	23.1	0.85	(0.61, 1.20)	.35	0.95	(0.69, 1.29)	.73
Indeterminate	7	1	14.3	0.13	(0.01, 1.47)	.10	0.11	(0.01, 1.11)	.06
Previously tested for HIV^a^									
No	223	35	15.7	REF					
Yes	2,029	561	27.7	1.87	(1.14, 3.07)	.01			
Received any information on HIV in past 6 months									
No	756	222	29.4	REF			REF		
Yes	1,499	374	25.0	0.70	(0.56, 0.86)	.001	0.72	(0.59, 0.89)	.002
FSW-CBO member									
No	1,650	474	28.7	REF			REF		
Yes	604	122	20.2	0.58	(0.46, 0.73)	<.001	0.73	(0.57, 0.92)	.01
Ever terminated a pregnancy									
No	1,348	312	23.1	REF			REF		
Yes	902	283	31.4	1.44	(1.17, 1.77)	<.001	1.41	(1.16, 1.72)	.001
Ever used emergency contraception									
No	1,896	406	21.4	REF			REF		
Yes	355	189	53.2	2.95	(2.45, 3.56)	<.001	2.70	(2.23, 3.26)	<.001
Intend to have children in the future									
Yes	1,452	345	23.8	REF					
No	761	238	31.3	1.00	(0.81, 1.25)	.99			
Does not know	42	13	31.0	0.63	(0.25, 1.60)	.33			
Number of clients in past month									
0–30	1199	343	28.6	REF					
31+	961	231	24.0	0.98	(0.80, 1.21)	.88			
Unknown	95	22	23.2	0.70	(0.36, 1.37)	.30			
Condom use during sex with clients									
Consistent/NA	1,749	434	24.8	REF			REF		
Inconsistent	503	162	32.2	1.23	(0.95, 1.59)	.11	1.00	(0.77, 1.29)	.98
Regular sex partner(s) in past year									
None	614	152	24.8	REF			REF		
Any	1,638	442	27.0	1.17	(0.93, 1.47)	.19	1.13	(0.91, 1.40)	.28
Any health-related stigma									
No	2,015	518	25.7	REF			REF		
Yes	237	78	32.9	1.49	(1.13, 1.96)	.004	1.29	(0.98, 1.69)	.07

Abbreviations: aPR, adjusted prevalence ratio; CBO, community-based organization; CI, confidence interval; FSW, female sex worker; NA, not applicable; PR, prevalence ratio; RDS, respondent-driven sampling; TOP, termination of pregnancy.

a Excluded from adjusted analyses due to collinearity with HIV status.

### Experience of Violence

Overall, 24.8% (559/2,251) of participants had experienced physical violence, and 32.6% (735/2,253) had ever been forced to have sex. Physical violence was most commonly perpetrated by clients (67.6%, 378/559), a uniformed officer (25.4%; 142/559), a boyfriend or husband (17.4%, 97/559), or another FSW (22.2%, 124/559). Sexual violence was most commonly perpetrated by clients (33.3%, 245/735), a stranger (29.3%, 215/735), or a boyfriend or husband (17.0%, 125/735).

## DISCUSSION

FSWs across 5 cities in Cameroon experienced a high burden of unintended pregnancy and TOP. Given the history of inconsistent or no condom use, nonbarrier contraceptive use is highly indicated to prevent unintended pregnancies and associated health consequences, but coverage among FSWs was low. These results remind us that the health needs of FSWs in Cameroon extend beyond HIV and underscore the importance of removing structural barriers as integral to optimizing coverage of health care services. The evidence suggests that community-based and FSW-focused services in Cameroon do not currently meet these broader needs. Comprehensive services, including access to client-centered, voluntary family planning counseling and services, are needed to effectively address FSWs' fertility and reproductive health needs.^54^

Although FSWs have a history of unintended pregnancy and termination of pregnancy coupled with inconsistent condom use, nonbarrier contraceptive use remains low.

Compared to other studies of FSWs, experience of TOP was similar or higher than observed across Southern Africa.^29^^,^^55^ TOP estimates among FSWs were twice as high as those reported among young women in urban areas or women seen in antenatal care (ANC) in other Cameroon-based studies.^38^^,^^40^ TOP performed in unsafe settings, even if supported by a health care worker, may lack appropriate counseling and information and miss opportunities to prevent future unintended pregnancy.^6^^,^^8^^,^^56^ Although this information was not collected here, among women with history of TOP in Cameroon, less than one-third reported currently using a non-barrier contraceptive and nearly one-quarter reported inconsistent condom use with clients. Subsequently, these women are likely at risk of future unintended pregnancies.

Findings from this study suggest that high burden of targeted violence in the context of criminalization experienced by FSWs may increase vulnerability to TOP.^16^ Links between violence and unintended pregnancy have also been reported in other settings,^57^ and associations between violence and HIV acquisition among FSWs are well-described.^14^^,^^16^^,^^58^ The relationships between violence and unintended pregnancy are likely similarly multifaceted.^58^^,^^59^ Violence and condom failure are commonly related,^15^^,^^46^ and past year condom failure was exceptionally common among this group of FSWs. In addition, it is possible that unintended pregnancy may itself trigger intimate partner violence.^60^ Given that physical violence is not factored into the conditions for legal abortion, FSWs experiencing physical violence leading to or arising from unintended pregnancy may perceive a lack of support to safely consider their options. Including access to emergency contraception and HIV postexposure prophylaxis as part of services for violence prevention and response could prevent unintended pregnancy and HIV acquisition resulting from sexual or physical violence. Programs that encourage social cohesion may help strengthen existing networks for violence coping and prevention mechanisms among FSWs.^16^^,^^61^ Uniformed officers have been common perpetrators of violence in this and other settings,^16^ suggesting the utility of training these officers in the public health outcomes of punitive enforcement as well as legal advocacy to overcome policy-level structural determinants in promoting greater protection and access to health and justice for FSWs.^47^^,^^61^

High burden of targeted violence experienced by FSWs may increase vulnerability to TOP.

Unintended pregnancy is largely preventable through effective nonbarrier contraceptives or consistent and correct condom use. FSWs in Cameroon were generally reliant on condoms for contraception, with limited use of nonbarrier methods and even lower use of more effective LARCs. This finding is consistent with the low use of LARCs in general across Cameroon,^34^ which despite government committment^62^ has not expanded at the same rate as observed in countries across eastern and southern Africa.^63^ We also identified differences in contraceptive access within Cameroon, with relatively higher access in Bamenda despite regional sociopolitical unrest. A similar finding has been previously reported in relation to ANC services in Cameroon and may be due to long-standing investment and resource mobilization in the region where Bamenda is located.^64^

Despite the reliance on condoms, most women did not report consistent condom use, particularly with nonpaying partners. There is substantial evidence from Cameroon and further abroad that pervasive structural factors, such as violence, stigmatization, price premiums for condomless sex, legal status, and policing, as well as social considerations, undermine FSWs agency to negotiate condom use consistently.^46^^,^^48^^,^^65^ Although current fertility intentions were not known, half of women were susceptible to pregnancy due to inadequate contraceptive use. Access to contraceptive choice and counseling is an essential part of sexual and reproductive health services.^54^ Expanding access to nonbarrier contraceptives and emergency contraception can reduce the risk of unintended pregnancy and related health, social, and economic consequences and provide women greater control over the timing and number of their children.^66^

Supplementary interventions targeting the social and structural influences on condom use are still needed to promote the comprehensive protection of dual-method use for HIV and STI prevention.^11^^,^^16^ Alternative user-controlled interventions for HIV prevention such as through pre-exposure prophylaxis may also benefit FSWs.^11^^,^^66^ Programs implementing single-purpose interventions such as contraception, pre-exposure prophylaxis, and antiretroviral therapy to FSWs can benefit from maintaining a comprehensive view of sexual and reproductive health. For example, proactively considering existing contraceptive mix and accessibility as pre-exposure prophylaxis is scaled up can maximize opportunities to prevent unintended pregnancies and counter potential changes in condom negotiation power or resource allocation.^66^^,^^67^ In addition to meeting the broader needs of FSWs, STI education, screening, prevention, and treatment programs can help prevent the potential STI-related risks associated with IUD use in the context of voluntary family planning services.^68^

Interventions targeting the social and structural influences on condom use are needed to promote the comprehensive protection of dual-method use for HIV and STI prevention.

With the exception of HIV testing, indicators of HIV service access—in particular FSW-oriented community services—were associated with substantially lower coverage of nonbarrier contraceptives. In comparison, membership in an FSW-oriented CBO was associated with higher recent HIV testing (data not shown). In Cameroon, most community-based facilities serving FSWs currently offer services fairly focused on HIV prevention, testing, and treatment support, with family planning often limited to counseling.^69^ Although these services appear to be an effective way to deliver HIV testing to FSWs,^70^ they may miss opportunities for contraceptive provision. Requiring women to access multiple service points to meet their sexual and reproductive health needs may further disincentivize contraceptive use and act as a structural barrier.^71^ This may be particularly relevant given there are already well-characterized barriers affecting FSWs' access to family planning through public clinics.^72^^,^^73^ Further qualitative research to assess personal considerations, barriers, and facilitators of voluntary contraceptive use among FSWs in Cameroon, including emergency contraception, could better inform local family planning delivery strategies and integration of services adapted to the needs and preferences of FSWs. Elsewhere, it has been demonstrated that FSW-led mobilization for HIV services through community drop-in centers has been associated with reductions in HIV incidence among FSWs in Tanzania.^70^ Expanding this community empowerment model for integrated family planning, STI, and HIV service delivery may represent an effective strategy to reduce unintended pregnancies and be responsive to critical health care needs.

Expanding community-based, FSW-led services to include integrated family planning, STI, and HIV service delivery may be an effective strategy to reduce unintended pregnancies among FSWs.

Although there is growing evidence that integrated HIV and family planning delivery in a single location facilitates contraceptive uptake,^71^^,^^74^^,^^75^ there are several considerations for implementation within existing community-based HIV services. There is empirical evidence that providing multiple contraceptive options improves uptake.^76^ Elsewhere, making longer-acting methods readily available to FSWs at a drop-in center alongside counseling on methods suited to a woman's needs and current fertility intentions significantly increased demand and uptake of effective contraceptives.^75^ However, the predominance of lower cadres of health care workers and peer outreach workers who often staff community-led FSW services may limit the number of staff with specific training and skills for contraception administration, especially when considering voluntary LARCs.^77^^,^^78^ Physical space with adequate privacy and space for an examination table required for LARC administration may also be limited in drop-in centers.^78^ Within the setting of community-based services, means to improve contraception availability and coverage may include task-sharing^79^ and collaborations with services already providing family planning or ANC to offer periodic clinics with outside staff within the community-based settings. Active, facilitated referrals may reduce the number of women falling through the service gap^80^ but may still pose barriers to some women. Lastly, not all FSWs in Cameroon are regularly accessing HIV testing^12^ and the majority were not associated with an FSW-focused CBO at the time of study. A single model of HIV and family planning integration is unlikely to reach all women, and considering alternative points of service access for multi-pronged, bidirectional approaches to integration and family planning service delivery may optimize coverage.^81^^,^^82^

As for many working cisgender women, FSWs are often mothers with ongoing fertility desires.^83^ Consequently, they would benefit from family planning services that are adaptive to changes in fertility intentions and contraceptive needs, including services for safer conception and pregnancy. Given pregnancy experience, including a sizeable proportion of pregnancies occurring after women began sex work, FSWs may benefit from greater links between FSW-oriented services and ANC. Approximately one-third of FSWs engaged in sex work during their last pregnancy, with potential for ongoing acquisition risks and resulting vertical transmission, especially if HIV is newly acquired in this period and missed by traditional approaches for preventing vertical transmission.^84^ Although the majority of women had sought ANC at last pregnancy, it is has been documented that FSWs as well as young, unmarried women commonly face stigma in ANC services and subsequently may avoid disclosure, thus limiting access to appropriate services.^85^^–^^87^ In our case, we identified associations between health care-related stigma and current use of nonbarrier contraception. Strengthening bidirectional collaborations between FSW-oriented services and ANC may foster more equitable access to quality and respectful family planning and ANC services for FSWs, including women living with HIV.

### Limitations

These findings are subject to several limitations. Indicators related to sexual and reproductive health were asked as part of a broader survey on HIV risk and vulnerability. There was limited space to comprehensively explore domains related to unintended pregnancy and TOP experiences. Because we did not assess current pregnancy intentions, we could not determine the current unmet need for contraception. Pregnancy outcomes were assessed based on lifetime experience rather than specific to each pregnancy, and contraception was assessed in a different timeframe based on current use. TOP was used as an indirect measure of unintended pregnancy due to biases associated with direct, retrospective measurement of unintended pregnancy.^51^ Some discrepancy is expected given that not all unintended pregnancies will result in TOP and TOP may also occur after planned pregnancies. With the exception of HIV status, all indicators were based on self-report and may be subject to social desirability and recall biases. However, despite social desirability biases, reported history of termination was high and reported use of current contraception low. Importantly, data collected were cross-sectional and causality cannot be inferred. Lastly, findings are based on FSWs recruited from 5 urban cities; access to contraception and TOP likely differs among women residing in rural areas, and findings cannot be generalized.

## CONCLUSION

In conclusion, experience with unintended pregnancy is common among FSWs in Cameroon, and women seeking TOP likely face risks associated with unsafe abortion given the restrictive legal environment. The ultimate direct and indirect impacts of TOP on health and mortality cannot be ascertained from these cross-sectional data, and longitudinal follow-up of women is needed to understand sexual and reproductive health outcomes that transcend HIV. The structural drivers of excess violence among FSWs remain a critical barrier to upholding the sexual and reproductive health and human rights of FSWs consistent with the United Nations Declaration of Human Rights. Reliance on condoms alone for preventing both HIV and pregnancy are failing the health needs of FSWs in Cameroon. Although the potential preventive impact of condoms has been substantial and continued promotion is critical, improved access to high-quality family planning counseling and a wider range of contraceptives, including nonbarrier and particularly long-acting contraceptives, is also necessary to improve client-centered care, promote informed choice, and reduce unintended pregnancies. Ultimately, mitigating structural and facility-level barriers to the coverage of high-quality, voluntary family planning services and method choice in FSW-focused community services represents a key strategy to overcome current barriers to access and move towards optimizing sexual and reproductive health outcomes among FSWs.

## Notes

**En français**:

**Besoins non satisfaits en planning familial et cas de grossesses non désirées chez les travailleuses du sexe en zone urbaine au Cameroun: Résultats d'une étude nationale transversale**

Les travailleuses du sexe (TS) au Cameroun ont des besoins insatisfaits en contraception efficace, et les cas de grossesses non désirées et d'interruptions de grossesse sont fréquents. La réduction des barrières à l'accès aux services de qualité de planning familial volontaire dans les services communautaires dédiés aux TS est une stratégie majeure dans la promotion des soins centrés sur le client, du choix éclairé, de la réduction des grossesses non désirées et l'amélioration de la qualité de vie des TS.

**RÉSUMÉ**

**Contexte:** Les besoins en contraception des travailleuses du sexe (TS) au Cameroun sont généralement non satisfaits, ce qui représente un risque élevé de grossesses non désirées. Les grossesses non désirées entraînent une série de résultats et, en raison de restrictions légales, les FSW recherchent souvent des avortements dangereux. Hormis la charge élevée du VIH, très peu d'informations relatives à la vie sexuelle et reproductive des TS au Cameroun sont connues.

**Méthodes:** De décembre 2015 à octobre 2016, des TS âgées de plus de 18 ans ont été recrutées dans 5 villes du Cameroun à l'aide d'un échantillonnage fondé sur les répondants. Des données transversales ont été recueillies à l'aide d'un questionnaire comportemental et du dépistage biologique du VIH/syphilis. Une approche modifiée et fiable de la régression de Poisson a été utilisée pour parvenir à une approximation des taux de prévalence ajustés (TPa) des IVG et l'utilisation en cours de contraceptifs non barrières efficaces.

**Résultats:** Des 2 255 TS (âge médian, 28 ans), 57,6% ont rapporté une grossesse non désirée et 40% une IVG antérieure. Au cours de l'analyse multi-variables, les antécédents d'IVG ont été associés à l'utilisation de méthodes contraceptives non barrières (TPa=1,23, 95% intervalle de confiance [IC]=1,07, 1,42); l'utilisation systématique de la contraception d'urgence (TPa=1,34, 95% IC=1.17, 1,55); >60 clients au cours du mois précédent (TPa=1,29, 95% IC=1,07, 1,54) contre £30; l'utilisation non systématique du préservatif avec les clients (TPa=1,17, 95% IC=1,00, 1,37); la violence physique (TPa=1,24, 95% IC=1,09, 1,42); et l'âge avancé. La majorité des femmes (76,5%) utilise le préservatif masculin comme contraception, mais seules 33,2% ont déclaré une utilisation constante du préservatif avec tous les partenaires. Au total, 26,4% des femmes ont déclaré qu'elles utilisent actuellement une méthode contraceptive non barrières, et 6,2% ont déclaré qu'elles utilisent une méthode contraceptive à longue durée d'action. Les antécédents d'IVG (TPa=1,41, 95% IC=1,16, 1,72) et l'utilisation systématique de la contraception d'urgence (TPa=2,70, 95% IC=2,23, 3,26) ont été associés à l'utilisation élevée de méthodes contraceptives dites non barrières. L'obtention récente d'informations sur le VIH (TPa=0,72, 95% IC=0,59, 0,89) et l'adhésion à une organisation à base communautaire pour les TS (TPa=0,73, 95% IC=0,57, 0,92) ont ééé associées à la faible utilisation des méthodes contraceptives non barrières.

**Conclusion:** Les cas de grossesse non désirées et d'VG sont fréquents chez les TS au Cameroun. Au vu de la faible utilisation des méthodes contraceptives non barrières et la non utilisation systématique du préservatif, les TS présentent un risque de contracter des grossesses non désirées de manière répétée. Une meilleure intégration des services de planning familial volontaire axés sur le client au sein des services communautaires du VIH permettraient de mieux appuyer la santé sexuelle et reproductive et les droits de l'homme des TS conformément à la Déclaration des droits de l'homme des Nations Unies.

**Peer Reviewed**

**Funding:** This research was generously supported by the U.S. President's Emergency Plan for AIDS Relief (PEPFAR) through the U.S. Agency for International Development (USAID) under the terms of the Continuum of prevention, care, and treatment of HIV/AIDS with Most at-risk Populations project. AB is supported by an Australian National Health and Medical Research Council (NHMRC) Early Career Fellowship. SB's and SS's efforts were funded in part by a grant from the National Institute of Nursing Research of the National Institutes of Health (R01NR016650) and the Johns Hopkins University Center for AIDS Research (CFAR). CFAR is an National Institute of Health (NIH)-funded program (P30AI094189) that is supported by the following NIH co-funding and participating institutes and centers: National Institute of Allergy and Infectious Diseases, National Cancer Institute, National Institute of Child and Human Development, National Heart, Lung, and Blood Institute, National Institute on Drug Abuse, National Institute of Mental Health, National Institute on Aging, Fogarty International Center, National Institute of General Medical Sciences, National Institute of Diabetes and Digestive and Kidney Diseases, and Office of AIDS Research. The content is solely the responsibility of the authors and does not necessarily represent the official views of USAID, PEPFAR, NIH, NHMRC, or other supporting agencies.
